# Preparation of Starch/Gelatin Blend Microparticles by a Water-in-Oil Emulsion Method for Controlled Release Drug Delivery

**DOI:** 10.1155/2014/829490

**Published:** 2014-04-29

**Authors:** Theeraphol Phromsopha, Yodthong Baimark

**Affiliations:** Biodegradable Polymers Research Unit, Department of Chemistry and Center of Excellence for Innovation in Chemistry, Faculty of Science, Mahasarakham University, Mahasarakham 44150, Thailand

## Abstract

Information on the preparation and properties of starch/gelatin blend microparticles with and without crosslinking for drug delivery is presented. The blend microparticles were prepared by the water-in-oil emulsion solvent diffusion method. Glutaraldehyde and methylene blue were used as the crosslinker and the water-soluble drug model, respectively. The blend microparticles were characterized by scanning electron microscopy (SEM), Fourier transform infrared (FTIR) spectroscopy, and UV-Vis spectroscopy. The functional groups of the starch and gelatin blend matrices were determined from the FTIR spectra. Blend microparticles with a nearly spherical shape and internal porous structure were observed from SEM images. The average particle size of the gelatin microparticles depended on the crosslinker ratio but not on the starch/gelatin blend ratio. The *in vitro* drug release content significantly decreased as the crosslinker ratio increased and the starch blend ratio decreased. The results demonstrated that the starch/gelatin blend microparticles should be a useful controlled release delivery carrier for water-soluble drugs.

## 1. Introduction

The development of biocompatible polymeric microparticles for controlled release drug delivery applications has been a subject of great interest for the past few decades [1–4]. Gelatin derived from collagen is a biodegradable and biocompatible natural polymer that has recently been extensively investigated as a biomaterial for use in biomedical applications such as tissue engineering [5] and drug delivery [6, 7]. The water-in-oil (W/O) emulsion-coacervation method has been widely used to prepare gelatin microparticles [6–8]. In this method, the W/O emulsion droplets of gelatin solution were crosslinked by crosslinking agents such as glutaraldehyde and genipin solutions. Starch is a nontoxic and biodegradable polysaccharide that has been widely investigated for biomedical [9] and pharmaceutical [10, 11] applications. The swelling and dissolution of both gelatin and starch microparticles have been controlled by chemical crosslinking [6–8, 10, 11]. However, the formation of crosslinked starch/gelatin blend microparticles as a drug delivery system has not yet been reported.

Our previous work has shown that the W/O emulsion solvent diffusion method was a potential method for preparing hydrophilic polymeric microparticles such as chitosan [4], starch [4, 11], and silk fibroin [12, 13]. In the present work, starch/gelatin blend microparticles containing a water-soluble model drug were prepared by the water-in-oil emulsion method. The influences of the glutaraldehyde-crosslinking and starch/gelatin blend ratio on microparticle characteristics and drug release profile were determined.

## 2. Materials and Methods

### 2.1. Materials

Gelatin powder was purchased from LabChem (Pennsylvania, USA). Water-soluble starch was provided by BDH Chemicals (Poole, UK). Glutaraldehyde solution (50% in water, 5.6 M) was purchased from Fluka (Steinheim, Germany). Methylene blue (98%) was used as a water-soluble model drug and purchased from Ajax Finechem (New South Wales, Australia). All chemicals were used without further purification.

### 2.2. Preparation of Drug-Loaded Blend Microparticles

The drug-loaded gelatin microparticles were prepared by the W/O emulsion solvent diffusion method. 2.0 mL of a 1% (w/v) gelatin aqueous solution was slowly added dropwise to 400 mL of 1% (w/v) Span80 (Merck, Pennsylvania, USA) in ethyl acetate (AR, Lab Scan, Samutsakorn, Thailand) under magnetic stirring at 900 rpm to prepare the non-crosslinked microparticles. The emulsification-diffusion process took 1 h. The methylene blue, the water-soluble model drug, was directly dissolved in the gelatin solution before microparticle preparation. The beaker was tightly covered with aluminum foil to prevent ethyl acetate from evaporation during the emulsification-diffusion step. The obtained microparticles were collected and rinsed with fresh ethyl acetate before drying in a vacuum oven at room temperature overnight. For crosslinking, the gelatin solution was crosslinked with glutaraldehyde for 4 h at room temperature under magnetic stirring before the addition of the water-soluble model drug and microparticle production.

Starch/gelatin blend microparticles were prepared from a starch/gelatin blend solution with the same conditions for the gelatin microparticles as described above. The 1% (w/v) starch and 1% (w/v) gelatin aqueous solutions were mixed under magnetic stirring for 30 min. The blend microparticles with starch/gelatin blend ratios of 20/80, 35/65, and 50/50 (w/w) were prepared. The starch/gelatin blend solution was crosslinked with a glutaraldehyde solution before the blend microparticle formation. The resulting microparticles were washed with 0.1 M glycine, followed by deionized water to eliminate the aldehyde residue [14] before being kept in a desiccator before characterisation of their properties and drug release testing.

### 2.3. Characterisation of Drug-Loaded Blend Microparticles

The morphology of the blend microparticles was analysed by scanning electron microscopy using a JEOL JSM-6460LV scanning electron microscope (SEM, Japan). The microparticle samples were sputter-coated with gold to enhance the surface conductivity before scanning. The average particle size of the blend microparticles was determined from several SEM images by counting a minimum of 100 particles using the smile view software (version 1.02). Chemical functional groups of the blend microparticles were determined by Fourier transform infrared (FTIR) spectroscopy using a Perkin-Elmer Spectrum GX FTIR spectrophotometer (USA). A resolution of 4 cm^−1^ and 32 scans was employed.

Drug loading of the microparticles was measured by dissolving the microparticles in 2% (w/v) NaOH solution before adjusting to pH 7 with HCl solution. The drug content was determined using a UV-Vis spectrophotometer (Lambda 25, USA) at 668 nm. According to a predetermined methylene blue concentration-UV-Vis absorbance standard curve, the methylene blue model drug concentration of the medium was obtained. Theoretical drug loading content (DLC_theoretical_) and actual drug loading content (DLC_actual_) were calculated from (1) and (2) and were used to measure the drug loading efficiency (DLE) as in (3). Consider the following:

(1) DLCtheoretical(%) =feed drug(mg)feed starch/gelatin+feed drug(mg)×100,

(2) DLCactual(%) =encapsulated drug(mg)resulting drug−loaded blend microparticles(mg)   ×100,

(3) DLE(%)=DLCactualDLCtheoretical×100.

### 2.4. *In Vitro* Drug Release Test

An* in vitro* drug release test was performed in a phosphate buffer solution (PBS, 0.1 M, pH 7.4) at 37°C under shaking. The drug-loaded blend microparticles (~20 mg) were suspended in 2 mL of buffer. At predetermined time intervals, 1 mL of release medium was withdrawn after centrifugation at 5,000 rpm for 5 min. Then 1 mL of fresh buffer was added to the original to maintain the total volume. The drug release was monitored by UV-Vis spectrophotometry at *λ*_max⁡_ = 668 nm and compared to a standard curve of drug solution. Cumulative drug release was calculated in terms of the ratio of the cumulative mass of the released drug at a given time against the initial drug loading in the microparticle sample.* In vitro* drug release tests were performed in triplicate (*n* = 3).

The data were expressed as mean ± SD. Statistical analysis was performed using a one-way analysis of variance (ANOVA).

## 3. Results and Discussion

### 3.1. Morphology and Size of Blend Microparticles

**Figure 1:**
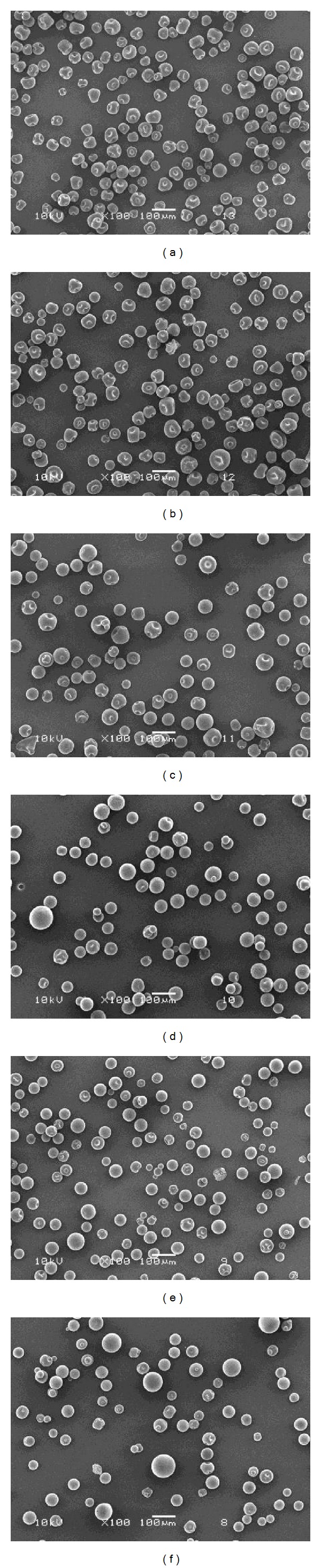
SEM images of drug-loaded gelatin microparticles (a) without crosslinking and crosslinked with (b) 12.5 g glutaraldehyde/100 g gelatin and (c) 25 g glutaraldehyde/100 g gelatin, and drug-loaded blend microparticles with starch/gelatin blend ratios of (d) 20/80, (e) 35/65, and (f) 50/50 (w/w) crosslinked with 25 g glutaraldehyde/100 g starch/gelatin. (All bars = 100 *μ*m.)

The morphology of the gelatin and blend microparticles with and without crosslinking was determined from SEM images as shown in Figure 1. All gelatin microparticles had deflated shapes and smooth surfaces with fine dispersibility (Figures 1(a)–1(c)). This may be due to them containing an internal porous structure. However, the gelatin microparticles crosslinked with 25 g glutaraldehyde/100 g gelatin, in Figure 1(c), were nearly spherical in shape.

**Figure 2:**
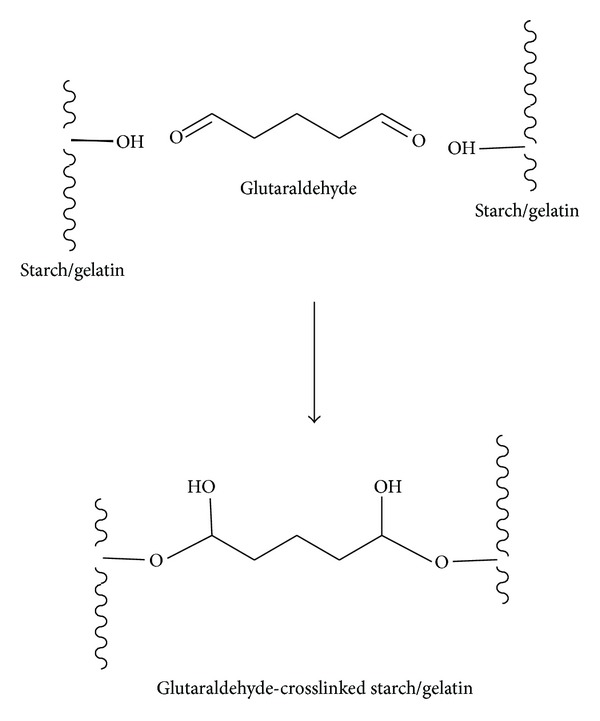
Possible crosslinking reaction of starch and gelatin by glutaraldehyde.

Figure 1 also shows SEM images of crosslinked blend microparticles prepared with different starch/gelatin blend ratios (Figures 1(d)–1(f)). It can be seen that they were nearly spherical in shape and had smooth surfaces. This indicates that the starch/gelatin blend ratio did not affect the morphology of the blend microparticles. The crosslinking occurred between the hydroxyl groups of starch/gelatin molecules and the aldehyde groups of glutaraldehyde [15, 16], according to the possible reaction shown in Figure 2.

**Figure 3:**
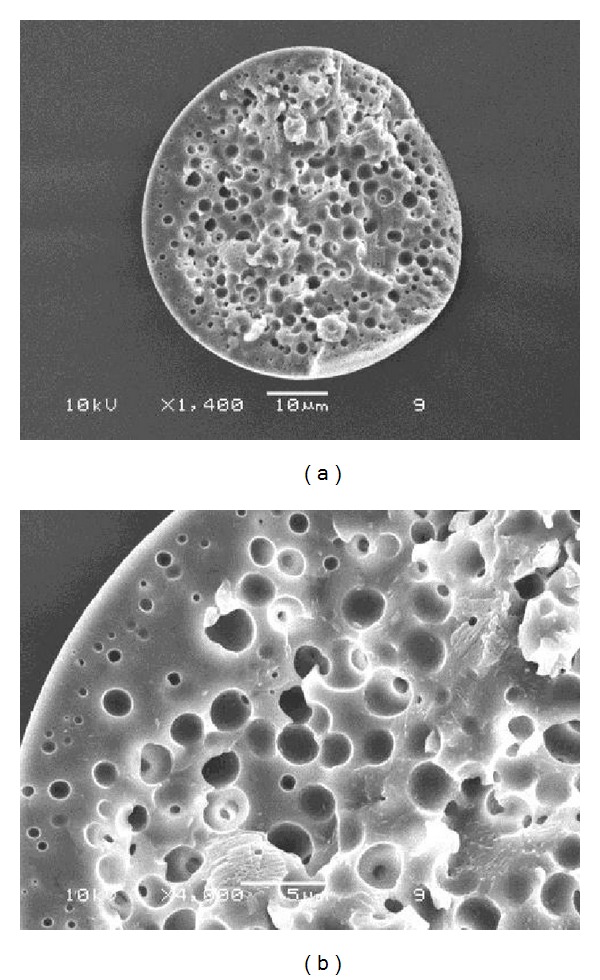
Internal morphology of broken drug-loaded 35/65 (w/w) starch/gelatin blend microparticles crosslinked with 25 g glutaraldehyde/100 g starch/gelatin with magnifications of (a) ×1,400 and (b) ×4,000. (Bars = 10 and 5 *μ*m for (a) and (b) images, resp.)

Internal morphology of the blend microparticles was observed from broken microparticles, an example of which is shown in Figure 3 for the 35/65 (w/w) starch/gelatin blend microparticles. The internal porous structures were detected throughout the microparticle matrix as shown in Figure 3(a) and magnified further in Figure 3(b). It is hypothesized that phase separation takes place in the W/O emulsion droplets during the emulsification-diffusion step before solidification of the microparticle matrix. A small amount of the ethyl acetate continuous phase, diffused into the W/O emulsion droplets, dispersed phase and starch/gelatin matrix, which was then rapidly solidified to induce the internal porous structure [17].

The average particle sizes of the blend microparticles measured from the SEM images are summarized in Table 1. It was found that the average size of the non-crosslinked gelatin microparticles was 62 *μ*m. The average particle sizes increased slightly as the gelatin was crosslinked with glutaraldehyde and the glutaraldehyde ratio was increased. This may be explained by the glutaraldehyde-crosslinking increasing the viscosity of the gelatin solution. The higher viscosity of the crosslinked gelatin solution induced larger W/O emulsion droplets before solidification of the microparticles. The average sizes of the crosslinked blend microparticles prepared with different starch/gelatin blend ratios were in the range from 87 to 98 *μ*m. The starch/gelatin blend ratio did not affect the average size of the blend microparticles.

### 3.2. FTIR of Blend Microparticles

**Figure 4:**
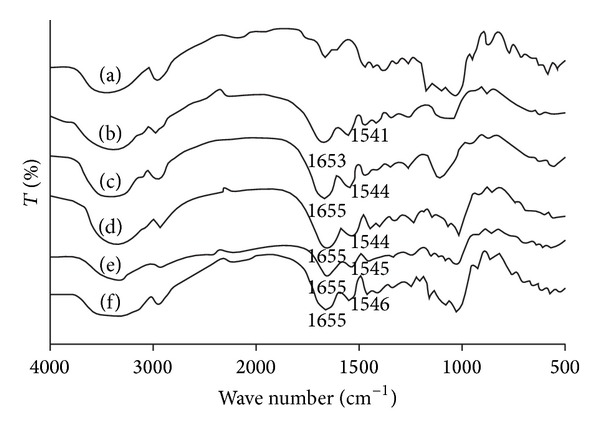
FTIR spectra of (a) starch and (b) gelatin and drug-loaded blend microparticles with starch/gelatin blend ratios of (c) 0/100, (d) 20/80, (e) 35/65, and (f) 50/50 (w/w) crosslinked with 25 g glutaraldehyde/100 g starch/gelatin.

FTIR spectra were used to confirm the chemical functional groups of the gelatin and starch as shown in Figure 4. For starch powder (Figure 4(a)), a broad band was observed in the range from 900 to 1200 cm^−1^ that is due to polysaccharides [18, 19]. The FTIR spectrum of the gelatin powder in Figure 4(b) exhibited absorption bands at 1653 cm^−1^ (amide I) and 1541 cm^−1^ (amide II). As would be expected, band intensities of the polysaccharide characteristics of the drug-loaded blend microparticles significantly increased and the amide characteristics decreased as the starch blend ratio increased as shown in Figures 4(c)–4(f). The FTIR results supported the fact that blend microparticles with various starch/gelatin blend ratios can be prepared.

### 3.3. Drug Loading and Drug Release of Blend Microparticles

The theoretical drug loading content (DLC_theoretical_) of the non-crosslinked and crosslinked microparticles calculated from the weights of the feed drug model and starch/gelatin as in (1) was 1.96%. The actual drug loading content (DLC_actual_) and drug loading efficiency (DLE) of the microparticles calculated from (2) and (3), respectively, are summarized in Table 1. The DLC_actual_ and DLE were in the ranges of 0.57 to 0.62% and 29 to 32%, respectively. The DLE of the methylene blue drug model was low. This may be due to diffusion out of the small model drug molecules, methylene blue, during microparticle solidification [11]. However, the larger model drug molecules, bovine serum albumin, exhibited high DLE value (~80%) in the polysaccharide-based microparticles [4]. The results of the drug loading suggested that the glutaraldehyde-crosslinking and starch/gelatin blend ratio did not affect the drug loading efficiency of the gelatin and blend microparticles. These microparticles with similar DLC_actual_ values were then used to study the influences of glutaraldehyde ratio and starch/gelatin blend ratio on drug release behaviours without the effect of drug concentration gap between the microparticle matrix and the released medium.

**Figure 5:**
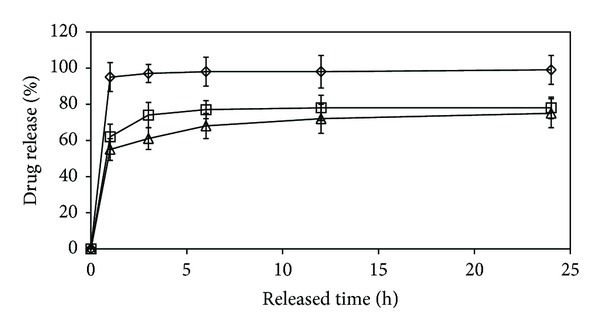
*In vitro* drug release profiles of drug-loaded gelatin microparticles crosslinked with different glutaraldehyde ratios. (*⋄*) non-crosslinked gelatin, (□) 12.5 g glutaraldehyde/100 g gelatin, and (Δ) 25 g glutaraldehyde/100 g gelatin.

The* in vitro* drug release of the methylene blue from the gelatin and blend microparticles was investigated in a phosphate buffer with pH 7.4 at 37°C for 24 h.  Figure 5 shows the drug release profiles of the gelatin microparticles as a function of the glutaraldehyde ratio. The non-crosslinked gelatin microparticles exhibited nearly complete drug release within 1 h (~95%) due to the fast dissolution of the gelatin microparticle matrix. The crosslinked gelatin microparticles showed a decrease in the methylene blue release over the whole period of time compared to the non-crosslinked gelatin microparticles as shown in Figure 5. The initial burst release within the first hour of release time was followed by a further sustained release that can be observed for the crosslinked gelatin microparticles. The mechanism of drug release consisted of two main steps: swelling-controlled and erosion-controlled steps [20]. The initial burst release of the drug model is due to the drug concentration gradient by matrix swelling. The slow drug release may occur due to surface erosion or dissolution of the microparticle matrix.

**Figure 6:**
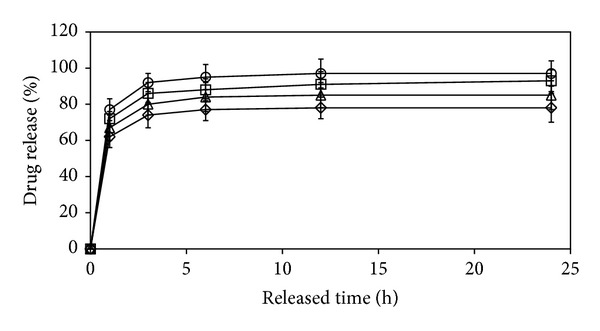
*In vitro* drug release profiles of drug-loaded blend microparticles prepared with starch/gelatin blend ratios of (*⋄*) 0/100, (Δ) 20/80, (□) 35/65, and (○) 50/50 (w/w) crosslinked with 25 g glutaraldehyde/100 g starch/gelatin.

The methylene blue release from the gelatin microparticles crosslinked with 12.5 g and 25 g glutaraldehyde/100 g gelatin at 24 h release time was about 78% and 74%, respectively. The glutaraldehyde-crosslinking can decrease the methylene blue release content. This may be explained by the crosslinked structure of the gelatin matrix inhibiting the drug release. For all the crosslinked starch/gelatin blend microparticles, a sustained drug release can be obtained as shown in Figure 6. For starch/gelatin blend ratios of 0/100, 20/80, 35/65, and 50/50 (w/w), the drug release levels at 24 h were 78%, 85%, 93%, and 97%, respectively. The level of drug release steadily increased as the starch blend ratio increased.

## 4. Conclusions

In this research, blending of starch and gelatin results in formation of nearly spherical microparticles which exhibit a promise to function as a carrier of water-soluble drug model, methylene blue. The drug-loaded microparticles prepared in this paper show sustained release of the drug model. The SEM images indicate that glutaraldehyde-crosslinking and starch blending enhance sphericality of the microparticle shape. The obtained microparticles slightly increase in their average particle size with increasing glutaraldehyde and starch blending ratios. The FTIR analysis of microparticles confirms the presence of starch and gelatin in the blend microparticles.

The drug loading efficiency of microparticles is not influenced by glutaraldehyde-crosslinking and starch blending. The extent of drug release expressed as percentage of drug release decreases with increasing glutaraldehyde ratio and decreasing starch blending ratio. These starch/gelatin blend microparticles have potential use as controlled release delivery carriers for water-soluble drugs.

## Figures and Tables

**Table 1:** Average particle size and drug loading of starch/gelatin blend microparticles.

Starch/gelatin (w/w)	Glutaraldehyde (g)/100 g starch/gelatin	Average particle size (*μ*m)	DLC_actual_ (%)	DLE (%)
0/100	—	62 ± 14	0.57 ± 0.08	29
0/100	12.5	84 ± 16	0.59 ± 0.12	30
0/100	25	92 ± 12	0.58 ± 0.09	30
20/80	25	87 ± 15	0.62 ± 0.14	32
35/65	25	95 ± 18	0.62 ± 0.06	32
50/50	25	98 ± 20	0.58 ± 0.12	30
